# Flooding performance evaluation of alkyl aryl sulfonate in various alkaline environments

**DOI:** 10.1371/journal.pone.0219627

**Published:** 2019-09-12

**Authors:** Lei Yan, Wei Ding

**Affiliations:** 1 School of Goverment, Peking University, Beijing, China; 2 College of Chemistry and Chemical Engineering, Northeast Petroleum University, Daqing, Heilongjiang, China; Qatar University, QATAR

## Abstract

Alkaline-Surfactant-Polymer (ASP) flooding is an efficient chemical enhanced oil recovery (EOR) method gaining popularity in the industry. In this paper, the characteristics of three flooding systems with alkyl aryl sulfonate surfactants and a weak alkali concentration, strong alkali concentration and no alkali concentration were investigated. The emulsification, interfacial tension, viscosity, stability, adsorption resistance as well as the oil displacement effect for the flooding systems and simulated oil of the fourth plant of the Daqing Oilfield were measured. The results show that the three alkyl aryl sulphonates surfactants have different emulsification indexes with the weak and strong alkali concentrations possessing the best and worst indexes at 67.00% and 55.17% respectively, and the combination of surfactant and no alkali concentration with an emulsification index of 63.03%. The interfacial tension between the three flooding systems and the simulated oil of the fourth plant of Daqing Oilfield gets as low as 10^−3^ mN/m, and reduces as far as 10^-4^mN/m in certain points detected, all with good anti-dilution performance. In terms of interfacial tension stability, the three flooding systems are seen to reach ultra-low interfacial tension within 90 days. For viscosity stability, the addition of a strong alkali and a weak alkali further hydrolyzes the polymer, leading to an initial rise in viscosity and viscosity retention rates above 80%. In terms of adsorption resistance, ultra-low interfacial tension occurs adsorption is reduced by five times for the strong and weak alkali systems, and reduced by four times for the alkali-free system. These results show that all three combination flooding systems have good adsorption resistance. In the evaluation of oil displacement effect, the average chemical flooding recovery rate (33.83%) of the weak alkali-surfactant-polymer (ASP) system is nearly three percent higher (31.34%) than that of the surfactant-polymer (SP) system, and over seven percent higher (26.71%) than that of the strong ASP system.

## Introduction

Alkali-Surfactant-Polymer (ASP) flooding is a new high-efficiency oil recovery technology, which was introduced in the 1980s. Currently, ASP flooding technology has made rapid progress, via several laboratory studies and pilot field tests in China[1]. ASP flooding system consists of alkali, surfactant, and polymer, and fully exerts the synergistic effect of the three components[2]. ASP flooding can not only increase the swept volume of the displacement phase but can also change the wettability of the rock through the adsorption of alkali and surfactant on the rock surface while forming an ultra-low interfacial tension of the oil-water two-phase flow to wash out the oil. The system is then emulsified by surfactants, thus enhancing oil recovery through emulsification[3]. Therefore, the EOR of ASP flooding mainly depends on two aspects: one is to improve the viscosity of the system and increase the swept volume; the other is to reduce the interfacial tension of oil and water and produce emulsification to improve oil displacement efficiency[4–5]. Successful practice of industrialized ASP flooding has been carried out on The Daqing Oilfield. Production shows that with regards to water flooding, ASP flooding can enhance oil recovery by more than 20%, which is over 10% higher than polymer flooding and is a feasible and effective method to enhance oil recovery[6–7]. The capillary number theory predicts that the oil recovery can be significantly enhanced only when the oil-water interfacial tension reaches ultra-low levels of 10^−3^ mN/m. The decisive standard for screening surfactants for oil displacement is dependent on whether surfactants can reduce the interfacial tension between oil and water to below 10^−2^ mN/m under a certain amount of use[8–10].

Polymers mainly contribute to the increased viscosity of an ASP flooding system. To obtain oil displacement systems with different viscosities, it is necessary to select different average molecular weights of polymer products and injection parameters according to reservoir conditions[11–12]. The formation of ultra-low interfacial tension between oil and water generally requires the synergy of the alkali and surfactant, depending on the type and amount of surfactant. The addition of alkali improves the ultra-low interfacial tension range and stability of the surfactant and reduces the adsorption of the surfactant. It is found that the addition of alkali, especially a strong alkali such as sodium hydroxide, does not only cause severe damage to the reservoir formation but also accelerates the scaling and corrosion of surface injection and production systems, thus leading to a significant increase in the cost of oil production in a field undergoing ASP flooding[13]. In order to avoid these defects, combination flooding has been developed from a strong-alkali ASP, weak-alkali ASP and SP systems.

In this paper, we study on the properties and oil displacement effects of an ASP flooding system prepared by using self-made alkyl aryl sulfonate surfactant, polymer, sodium hydroxide, sodium carbonate, and a synergist.

## Materials and instruments

The alkyl aryl sulfonate used in the study (XWY- I, XWY- II, XWY- III are types of strong alkali, weak alkali, and alkali-free respectively which were produced by Xinweiyuan Chemical Co., Ltd. in the Heilongjiang province), has effective content of 50 ±2 wt.%. The polymer, polyacrylamide has a relative molecular weight of 1.9 × 10^7^ with a solid content of 90 wt.% and is produced by Daqing Refining and Chemical Company. The simulated oil, with a viscosity of 10 mPa·s at 45°C, is prepared with aviation kerosene and the crude oil, dehydrated and degassed from the fourth plant of the Daqing Oilfield. The solvent is reinjected sewage water from the fourth plant of the Daqing Oilfield filtered through a 0.2 μm micropore to remove impurities. The remaining materials are pure analytical reagents.

The list of instruments employed in the study include the TX-500C full-range Spinning Drop Interfacial Tensiometer produced by Beijing Shengweijiye Technology Co., Ltd. was used to determinate the interfacial tension between the combination flooding system and the simulated oil. HT-Ⅱautomatic mixer produced by Jiangsu Hai'an Scientific Research instrument factory was used in the stage of neutral reaction. Turbiscan stability analyzer produced by Formulation Company of France, the T10 high speed disperser produced by IKA Company of Germany, DV-Ⅱ Brinell viscometer produced by Brookfield Company in the United States, 2000 series constant speed and constant pressure ISCO pump, a standard digital pressure gauge, HW- 4A thermostat, vacuum pump, DJYZ-58 pressure sensor, magnetic agitator and an F1104N electronic balance were used in the determination for emulsification stability and the evaluation of oil displacement effect. Other accessories include a valve, intermediate container, dead plug, nylon pipeline, tee, and cross. The experimental core used in the oil displacement test possessed a gas permeability measured at about 300 × 10^−3^ μm^2^ with a specification of 4.5 × 4.5 × 30 cm.

## Experimental methods

### Determination of a comprehensive emulsification index

The emulsification index is a physical quantity that characterizes the emulsification performance of the oil displacing agent[14]. It is obtained from the square root of the product of the emulsifying power and the emulsion stability and is measured in percentage.

### Determination of emulsifying power

Emulsifying power (f_e_) refers to the ability of surfactants to emulsify crude oil per unit mass. It is expressed by the ratio of the oil content of the emulsified phase to the initial oil content with its method of determination detailed in publications. First, the mixture of 50 ml simulated oil and 50ml combination flooding solution was stirred by the T10 disperser for 10 minutes (the simulated oil was excessive compared to the combination system) at a temperature of 45°C and rotating speed 10000r/min. This led to the formation of the emulsion and oil phase. Subsequently, the emulsion was separated from the mixture and contained oil extracted using petroleum ether. Finally, the optical density of the extract was measured, and the volume of emulsified oil in the combination flooding system obtained by comparison to the standard curve. The emulsification power (f_e_) is the percentage of the oil extracted in the emulsified phase and the total amount of the emulsified oil.

### Determination for emulsification stability

A 3 ml volume of simulated oil and 7 ml of surfactant in aqueous solution were put into a 20 ml piston cylinder. The mass fraction of surfactant was 0.3 wt.% and the rest consisting of the field sewage mentioned above. The mixture was incubated at a simulated formation temperature of 45°C for 30 minutes in a constant temperature oven. The piston was compressed and released 150 times to allow full emulsification of the simulated oil and was placed in the constant temperature oven again. After 1 hour, the volume of deposited water (V_w1_) was recorded from which the water evolution rate (S_w1_) and the emulsifying stability (S_te_) of surfactant were calculated according to Eqs (1) and (2) respectively.

$$S_{w1}=\frac{V_{w1}}{V_{2}}\times 100\%$$ (1)

$$S_{te}=1-S_{w1}$$ (2)

where, V_2_ is the volume of the original surfactant solution in ml.

The emulsification index is calculated by Eq (3).

$$S_{ei}=\sqrt{f_{e}∙S_{te}}$$ (3)

### Determination of interfacial tension

The interfacial tension between the combination flooding system and the simulated oil was measured using the TX-500C Spinning Drop Interfacial Tensiometer. The oil droplets were injected into the closed sample tube containing the surfactant solution to be tested. The sample tube was then placed in the interfacial tensiometer with a set rotational speed and a temperature of 45°C. During the experiment, photographs were taken automatically at 20 minutes intervals. After 2 hours, the diameter and length of oil droplets were measured from the photographs. The aspect ratio of the droplets is expected to be greater than 4, and the interfacial tension is recorded as equilibrium interfacial tension.

### Evaluation of oil displacement effect

1. The flooding schemes applied to the experimental Bailey core of dimensions 4.5 × 4.5 × 30 cm are detailed in Table 1.
2. The core was inserted into the core holder and connected to a vacuum for 4 hours preventing gas leakage. The pore volume was measured following re-injection of the sewage water into the saturated field.
3. The core was placed in a 45°C incubator for 15 hours, subsequently saturated with crude oil and heated in a 45°C incubator for 20 hours.
4. Water flooding was conducted at a displacement rate of 0.3 ml/min with the water flooding recovery calculated when the moisture content at the outlet of the model reaches 98%.
5. The chemical flooding slug was injected and flooded with water until the model outlet water content reaches 98%.

**Table 1:** Flooding schemes.

name	ASP flooding slug	Experimental scheme
Scheme 1	Polymer (1550 mg / L) + 0.4 wt.% synergist + 0.3 wt.% alkali-free system	Water flooding +0.3 PV SP flooding (40 cp) +0.2 PV polymer flooding (40 cp) + subsequent water flooding
Scheme 2	Polymer (1700 mg / L) + 0.3 wt.% surfactant + 1.2 wt.% weak alkali	Water flooding +0.3 PV weak alkali ASP flooding (40 cp) +0.2 PV polymer flooding (40 cp) + subsequent water flooding
Scheme 3	Polymer (1820 mg/L) +0.3wt.% surfactant + 1.2 wt.% strong alkali	Water flooding +0.3 PV strong alkali ASP flooding (40 cp) +0.2 PV polymer flooding (40 cp) + subsequent water flooding

## Results and discussion

### Determination of emulsification index

The concentration of fixed alkali was 1.2 wt.% (mass fraction), and the concentration of surfactant was 0.3 wt.% (mass fraction) (no alkali type, only added synergist, with mass fraction 0.4 wt.%). The stability of emulsions prepared by different alkyl aryl sulfonate (XWY- Ⅰ, XWY- Ⅱ, XWY- Ⅲ) was investigated with results shown in Table 2.

**Table 2:** Emulsification index of different flooding systems.

Flooding system	*S*_*te*_ (%)	*F*_*e*_ (%)	*S*_*ei*_ (%)
1.2 wt.% NaOH + 0.3 wt.% strong alkali system XWY-I	73.22	41.57	55.17
1.2 wt.% Na_2_CO_3_ + 0.3 wt.% weak alkali system XWY-II	94.21	47.65	67.00
0.4 wt.% synergist +0.3 wt.% alkali-free system XWY-III	86.58	45.89	63.03

For the three alkyl aryl sulfonates, the emulsification indexes occur in decreasing order of 67.00%, 63.03% and 55.17% for the weak alkali type (XWY-Ⅱ), strong alkali type (XWY-Ⅰ) and alkali-free type (XWY-III) respectively (Table 2). The three alkyl aryl sulfonates have different average molecular weights and molecular weight distributions. The weak alkali type has an average molecular weight of 460, and the molecular weight distribution is an inverse normal distribution[15]. The low molecular weight component with a short carbon chain and the high molecular weight component with a long carbon chain have a relatively even distribution and can be evenly arranged at the oil-water interface, thereby have a good emulsifying performance. The strong alkali type has an average molecular weight of 430 and increased molecular weight distribution, that is, the high molecular weight components with longer carbon chain are more than the low molecular weight components with shorter carbon chains. While the components with shorter carbon chains are easier to adsorb preferentially at the oil-water interface for emulsification, the high molecular weight components with very long carbon chains are easier to dissolve in the oil phase and thus do not emulsify readily. As a result, its emulsifying performance is the worst among the three surfactants. The average molecular weight of the alkali-free type is 480 and requires the addition of a certain amount of synergist to attain ultra-low interfacial tension. Its molecular weight distribution is evenly distributed, that is, the components of the high, medium and low molecular weights in the formulation are equally distributed. Experiments show that its emulsification index lies between the strong alkali and the weak alkali emulsification indexes.

### Interfacial tension and stability of combination flooding system

#### Determination of interfacial tension of combination flooding system

Measured interfacial tension between the three composite flooding systems and the simulated oil is given in Tables 3, 4 and 5.

**Table 3:** Interfacial tension between strong alkali ASP system and simulated oil.

Detection point (*w/w*) XWY-Ⅰ/NaOH	Rotating speed/ (r/min)	Polymer concentration / (ppm)	Test time (min)					
			20	40	60	80	100	120
			IFT×10^−3^ (mN/m)					
0.05/0.6	4750	1000	7.10	4.36	3.63	2.18	2.18	1.95
0.05/1.0	3300	1000	1.75	1.59	1.16	1.03	1.16	6.28
0.20/0.8	4750	1000	5.94	4.60	3.16	2.30	1.62	5.47
0.20/1.2	3300	1000	5.20	5.20	4.48	4.15	4.48	3.90
0.30/1.2	3300	1500	6.15	5.05	5.82	5.43	5.43	4.76

**Table 4:** Interfacial tension between weak alkali ASP system and simulated oil.

Detection point (*w/w*) XWY-Ⅱ/Na_2_CO_3_	Rotating speed/ (r/min)	Polymer concentration / (ppm)	Test time (min)					
			20	40	60	80	100	120
			IFT×10^−3^ (mN/m)					
0.05/0.6	4750	1000	18.4	9.45	6.09	4.76	2.99	1.78
0.05/1.0	3300	1000	1.75	1.16	1.43	1.93	3.23	3.50
0.20/0.8	4750	1000	12.1	6.95	5.02	4.60	4.60	5.02
0.20/1.2	3300	1000	2.84	1.99	1.32	0.95	0.42	3.30
0.30/1.2	3300	1500	8.81	7.79	5.05	4.62	3.58	3.01

**Table 5:** Interfacial tension between SP system and simulated oil.

Detection point(*w/w*) XWY-III/ synergist	Rotating speed/ (r/min)	Polymer concentration / (ppm)	Test time (min)					
			20	40	60	80	100	120
			IFT×10^−3^ (mN/m)					
0.05/0.4	4750	400	44.9	21.6	13.8	6.58	5.18	2.70
0.10/0.4	4750	400	16.2	4.00	1.48	0.84	0.50	0.41
0.20/0.4	4750	800	16.4	9.99	8.08	5.02	4.21	3.49
0.30/0.4	4750	1200	18.2	6.31	5.38	3.80	3.42	4.12

In oilfield testing practice, the methods of low alkali (alkali dosage < mass fraction 1.0%) with high rotation speed and high alkali (alkali dosage ≥ mass fraction 1.0%) with low rotation speed are adopted. It can be seen from Table 3 that the equilibrium interfacial tension of all five detection points in the strong alkali ASP system reached ultra-low levels in the order of 10^−3^ mN/m. Under the condition of low alkali and low surfactant concentration, the lowest equilibrium interfacial tension is 1.95 × 10^−3^ mN/m, which indicates that the strong alkali ASP system has good dilution resistance.

The same test method was adopted for the weak alkali ASP system and the strong alkali ASP system. It can be seen from Table 4 that the equilibrium interfacial tensions of all five detection points have reached ultra-low interfacial tension in the order of 10^−3^ mN/m, some extending to the order of 10^−4^ mN/m. Under the condition of low alkali and low surfactant concentration, the lowest equilibrium interfacial tension is 1.78 × 10−3 mN/m, which indicates that the weak alkali ASP system also has good dilution resistance.

For the SP system, the equilibrium interfacial tension of all five detection points also exceeds the ultra-low interfacial tension levels in the order of 10^−3^ mN/m, reaching the order of 10^−4^ mN/m in some instances (Table 5). In the case where the surfactant concentration is 0.1 wt.%, the synergist dosage is 0.4 wt.%, and the equilibrium interfacial tension is at least 4.10×10^−4^ mN/m, indicating that the SP system also has certain anti-dilution performance.

#### Stability of combination flooding system

**Fig 1:**
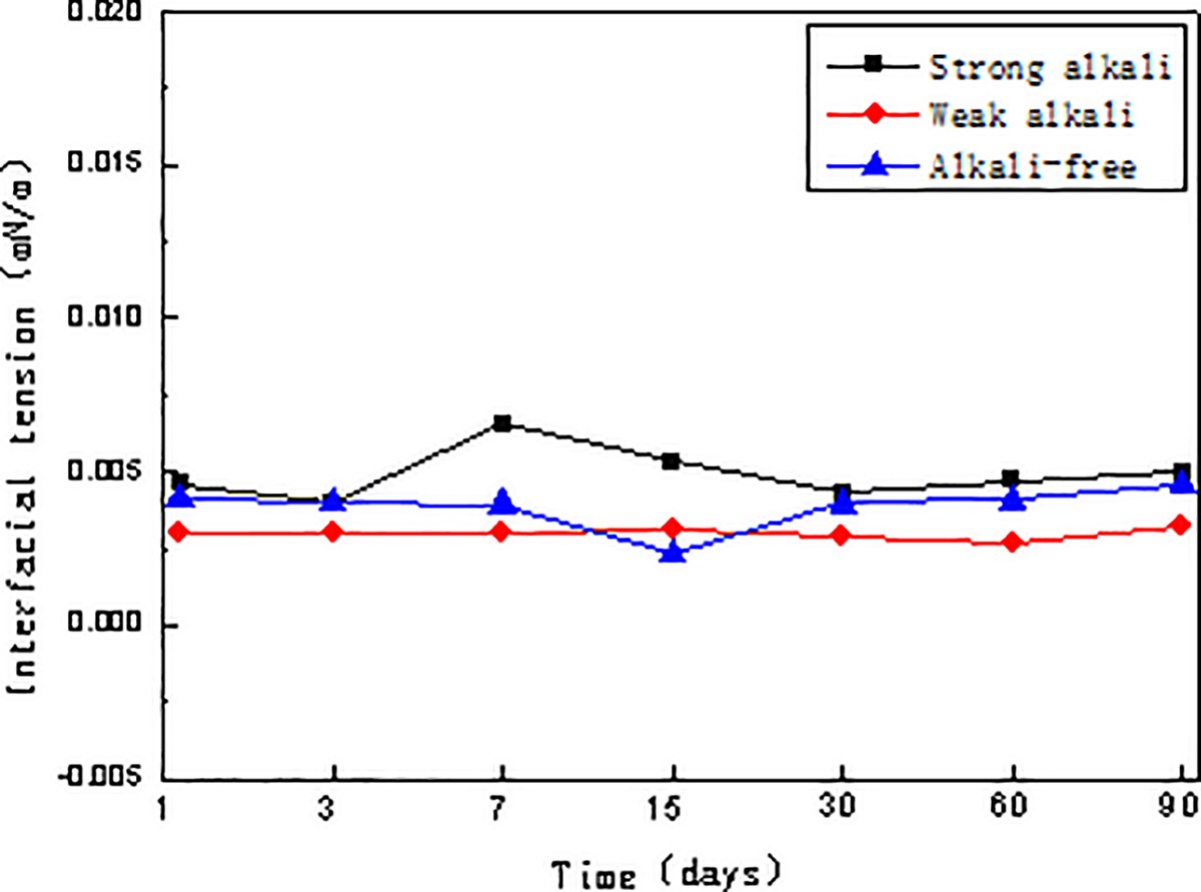
Interfacial tension stability curves of three kinds of combination flooding systems.

**Fig 2:**
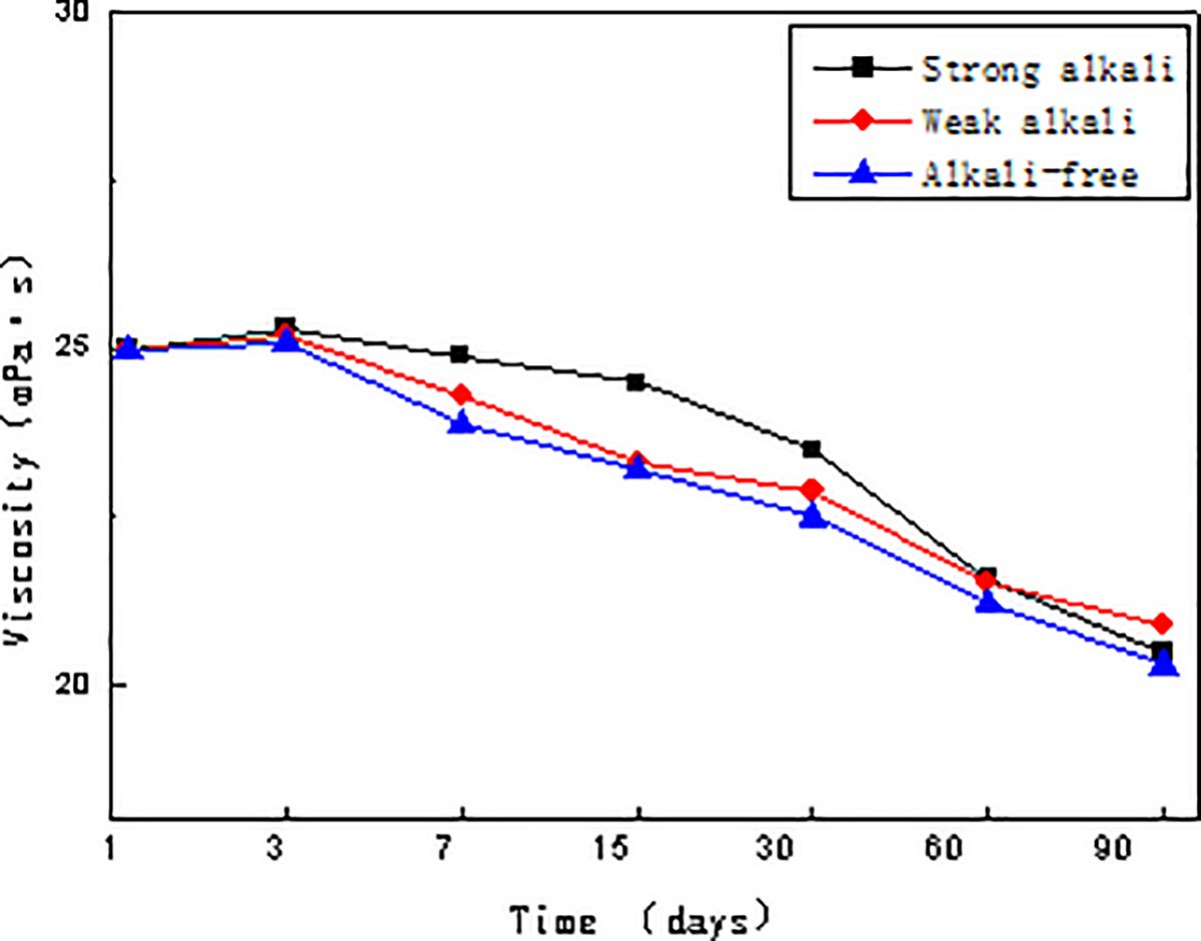
Viscosity stability curves of three kinds of combination flooding systems.

The combination system of alkali of 1.2 wt.% concentration (adding a synergist of 0.4 wt.% concentration in the SP system), alkyl aryl sulfonate of 0.3 wt.% concentration and viscosity 25mPa·s was prepared indoors. Results from the interfacial tension and viscosity stability tests conducted at different times (on day 1, 3, 7, 15, 30, 60 and 90) are represented in Figs 1 and 2.

In Figs 1 and 2, all three combination flooding systems attain good interfacial tension stability within 90 days and achieve ultra-low interfacial tension. Comparing viscosity stability trends, we can see the addition of the strong alkali and weak alkali further hydrolyzes the polymer, resulting in an initial rise of the viscosity. Thus, the viscosity retention rates of the three systems are all above 80%[16].

#### Adsorption resistance of combination flooding system

**Fig 3:**
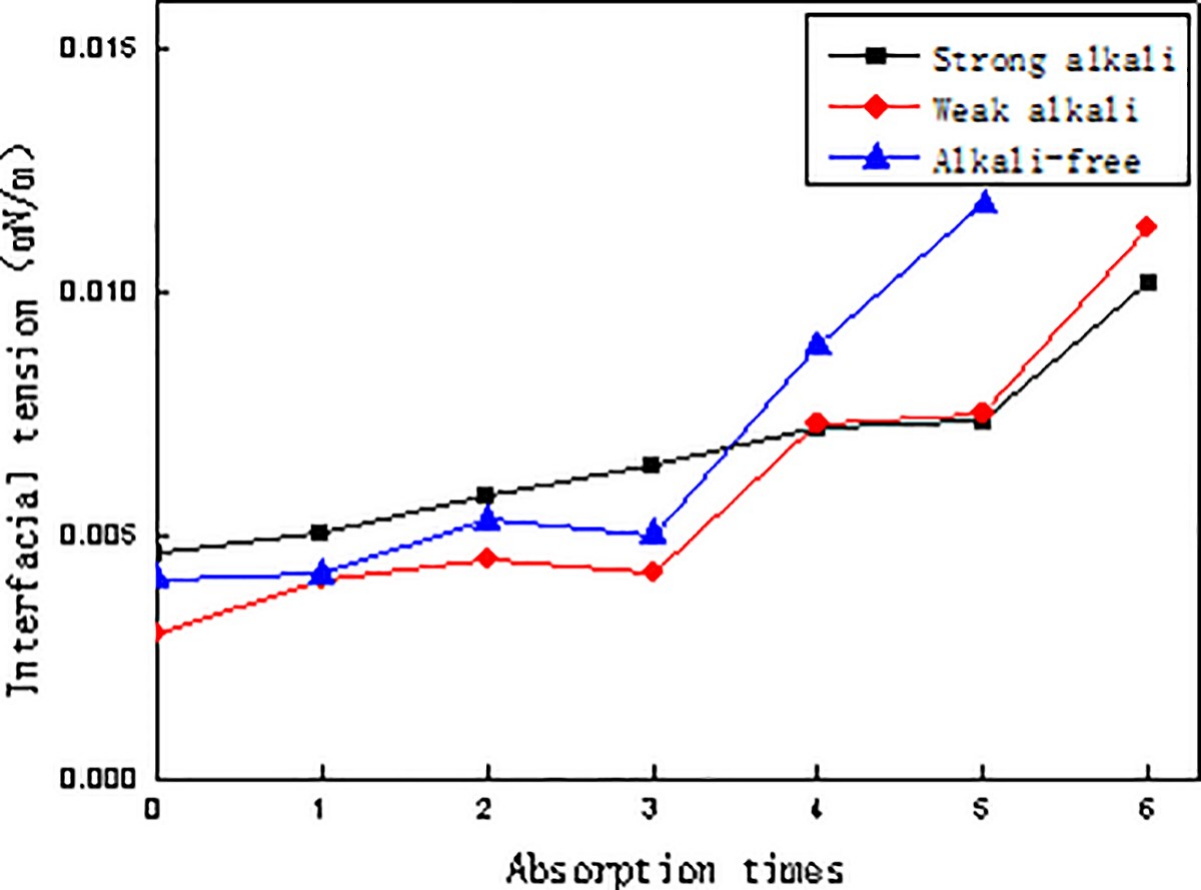
Adsorption interfacial tension curves of three kinds of combination flooding systems.

The solution of the composite system was prepared following a similar method as the solution before. Natural oil sand (80–120 mesh) was mixed at a scale of 9:1, according to the ratio of the combination system to oil sand and oscillated at 45°C for 24 hours. After centrifugal separation, part of the supernatant was taken to determine the interfacial tension of the system. The remaining supernatant and the new oil sand were again mixed in the ratio 9:1, followed by the next adsorption experiment under the same conditions. The above experimental steps were repeated continuously until the interfacial tension reached 10^−2^ mN/m. The results are depicted in Fig 3.

The data in Fig 3 shows that the interfacial tension of the strong alkali ASP system and the weak alkali ASP system can still reach the ultra-low level of 10^-3^mN/m after five adsorption times, indicating that the anti-adsorption properties of the two are excellent. However, the interfacial tension of the SP system can only reach the order of 10^−2^ mN/m after four adsorption times, indicating that its anti-adsorption property is poor.

### Oil displacement effect of combination flooding systems

For each combination flooding system, oil displacement experiments were conducted on two Bailey cores under the same conditions and according to the schemes in Table 1. The average value of each was taken as the evaluation of the EOR effect of combination flooding.

Table 6 shows the results of oil displacement tests following injection of the core with different combination flooding systems.

**Table 6:** Statistical results of oil displacement test.

Experiment scheme	Core number	Permeability to water	Porosity	Oil saturation	Water flooding recovery	Chemical flooding recovery	Chemical injection slug recovery	Subsequent water flooding recovery	Total recovery
		(mD)	(%)	(%)	(%)	(%)	(%)	(%)	(%)
Scheme 11	1811–1	204	20.54	63.59	37.10	30.84	20.52	10.32	67.94
Scheme 21	1811–2	204	20.46	65.10	38.10	32.93	22.00	10.93	71.03
Scheme 31	1811–3	215	20.59	64.85	37.41	27.10	20.02	7.08	64.51
Scheme 12	1811–4	201	19.64	67.90	37.05	31.83	19.71	12.12	68.88
Scheme 22	1811–5	150	20.19	64.55	37.81	34.73	21.67	13.06	72.54
Scheme 32	1811–6	139	20.06	63.05	38.28	26.31	19.08	7.23	64.59

As can be seen from Table 6, the oil displacement effects of the weak alkali ASP flooding, schemes 21 and 22, are better than that of the SP system schemes 11 and 12 and the strong alkali ASP flooding, schemes 31 and 32. The average chemical flooding recovery of weak alkali ASP flooding (33.83%) is nearly three percentage points higher than that of the SP flooding (31.34%) and seven percentage points higher than that of strong alkali ASP flooding (26.71%). Regarding the equilibrium interfacial tension, the SP flooding is 4.12×10^−3^ mN/m, the weak alkali ASP flooding is 3.01×10^-3^mN/m, and the strong alkali ASP flooding is 4.76. ×10^−3^ mN/m. All of them meet the requirements of ultra-low interfacial tension of surfactants for oil displacement. The weak alkali ASP flooding system not only reaches ultra-low interfacial tension but also has a good emulsifying ability. The recovery of the strong alkali ASP flooding during the subsequent water injection was significantly lower than both the weak alkali ASP and SP flooding, due to its weak emulsification.

**Fig 4:**
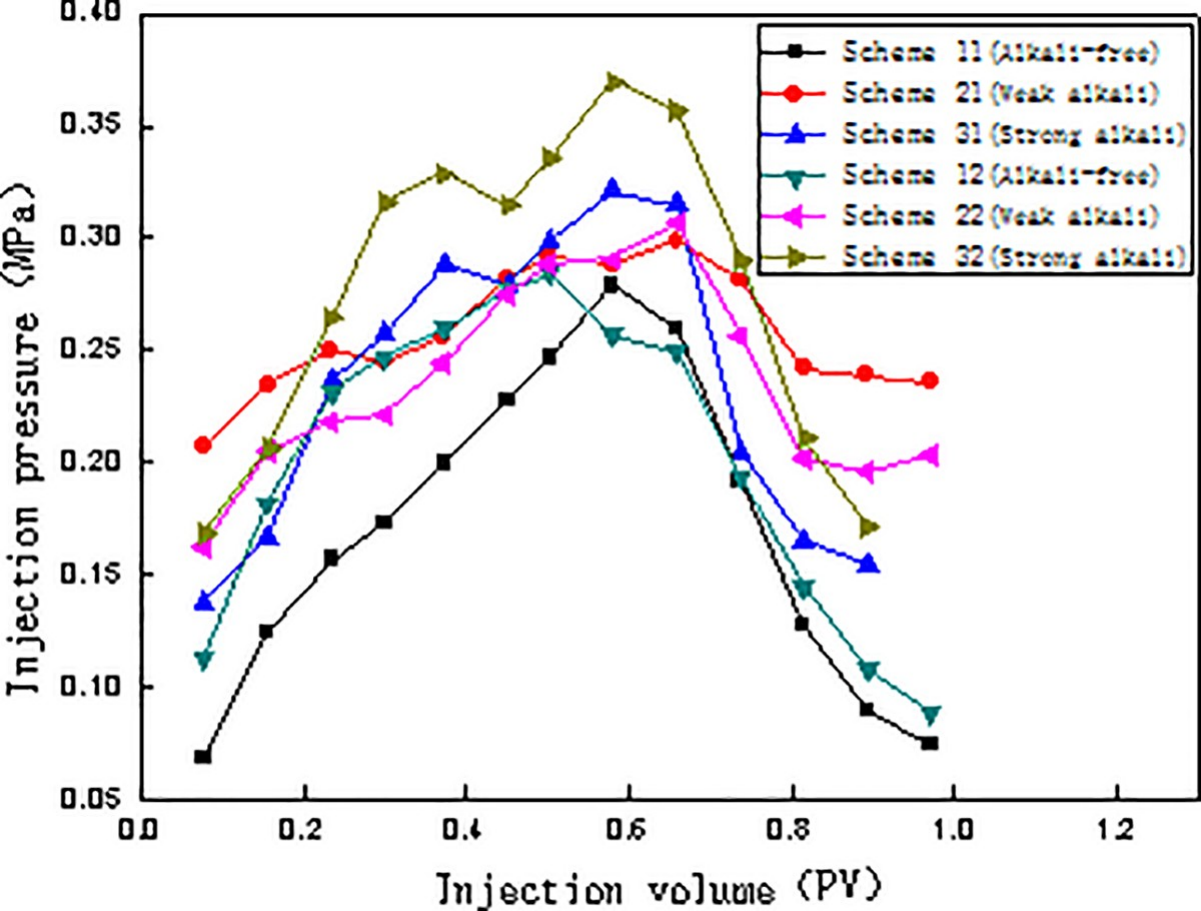
Relationship between injection pressure and injection volume for different flooding systems.

**Fig 5:**
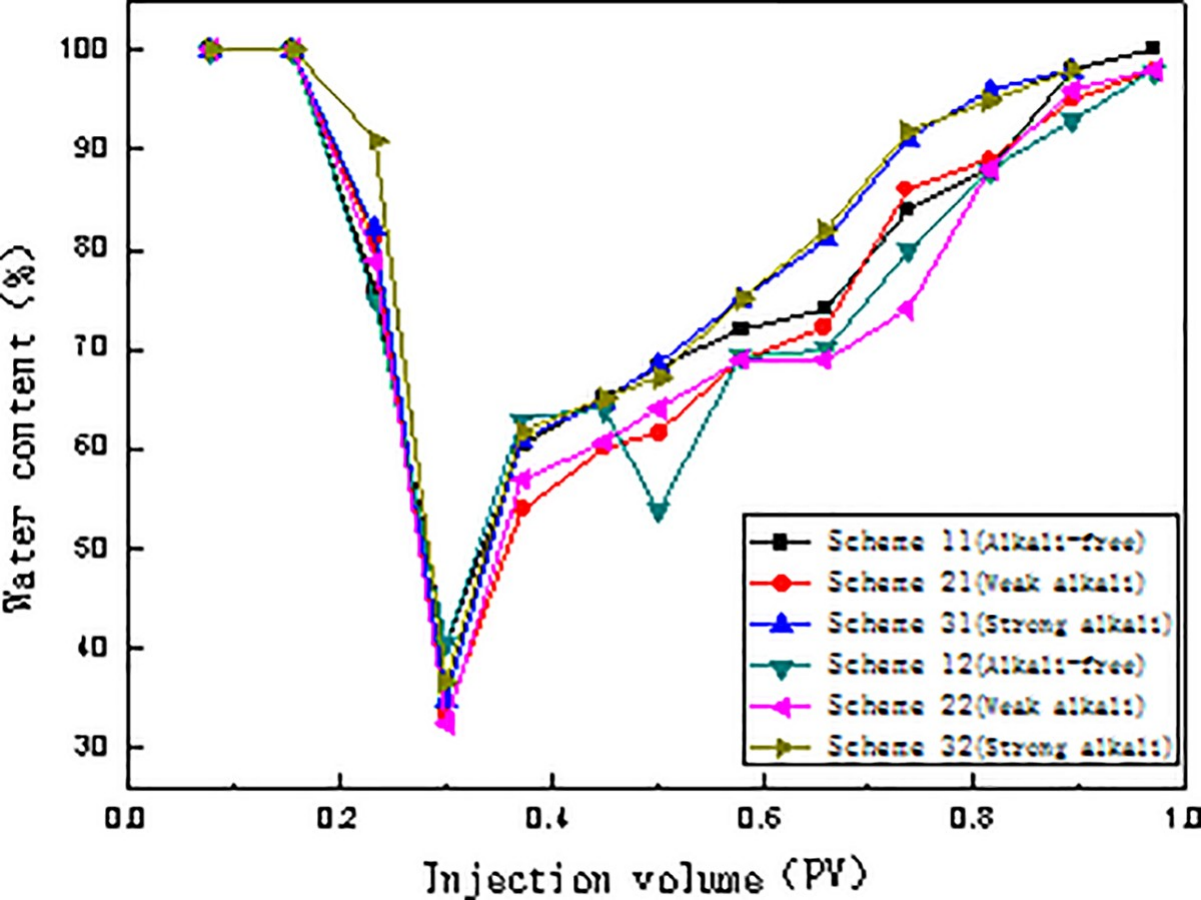
Relationship between water content and injection volume of different flooding systems.

**Fig 6:**
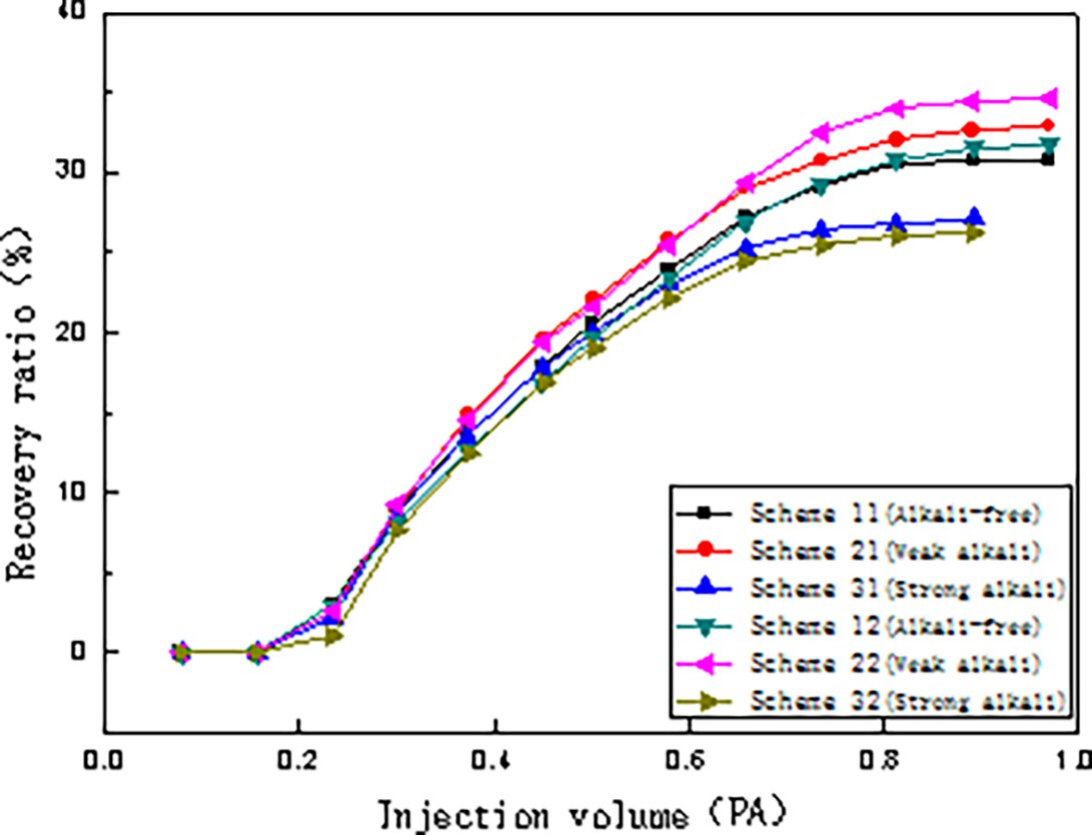
Relationship between recovery ratio and injection volume of different flooding systems.

The relationships between injection pressure and injection volume, water content and injection volume, and oil recovery and injection volume for the six schemes are presented in Figs 4, 5 and 6, respectively.

#### Relationship between injection volume and injection pressure

During the displacement experiment, the injection pressure first increases, then decreases significantly in the subsequent water flooding stage (Fig 4). In schemes 11, 12, 31 and 32, the injection pressure decreased significantly in the subsequent water flooding stage. In schemes 21 and 22, however, the injection pressure decreased slightly, indicating that the emulsification contributes to the injection pressure. Considering the perspective of high pressure, the injection pressure of the strong alkali ASP system has the highest relative magnitude, followed by that of weak alkali ASP system, while the injection pressure of the SP system is the lowest. The results show that the injection pressure certainly has an effect on the chemical flooding recovery. Too high or too low injection pressure does not achieve the best oil displacement effect. The injection pressure of the weak alkali ASP system is relatively average, thereby having the highest chemical flooding recover.

#### Relationship between injection volume and water content

Compared to the strong alkali ASP and SP systems, the weak alkali ASP system has the lowest water content and most extended period of low water content (Fig 5). Thus, the weak alkali ASP system has the best oil displacement effect. The equilibrium interfacial tension of the SP system is 4.12×10^−3^ mN/m, and is 3.01×10^−3^ mN/m and 4.76×10^−3^ mN/m for the weak alkali and strong alkali ASP systems respectively. Among the three systems, the weak alkali ASP has the lowest interfacial tension and best emulsifying ability, resulting in a long low water content period and relatively high chemical flooding recovery.

#### Relationship between injection volume and recovery ratio

In Fig 6, all three flooding systems demonstrate significant enhancement of the chemical flooding recovery with the weak alkali ASP flooding the highest, followed by SP flooding, and chemical flooding recovery of strong alkali ASP flooding the least.

## Conclusions

1. The weak alkali type (XWY-Ⅱ) possesses the best emulsification index of all three alkyl aryl sulfonates at 67.00%, while the strong alkali type (XWY-Ⅰ) has the worst with only 55.17% and the alkali-free type (XWY- Ⅲ) has an emulsification index of 63.03%.
2. Ultra-low interfacial tension between the three flooding systems and the simulated oil of the fourth Plant of the Daqing Oilfield occurs in the order of 10^−3^ mN/m, decreasing further to the order of 10^−4^ mN/m in certain detection points. In addition, all three combination flooding systems have good dilution resistance. The weak alkali ASP flooding system has the lowest equilibrium interfacial tension, followed by the SP flooding system, and the strong alkali ASP flooding system with the highest equilibrium interfacial tension.
3. All three combination flooding systems have good interfacial tension stability and can reach ultra-low interfacial tension within 90 days. Evaluation of viscosity stability shows that the polymer is further hydrolyzed by the addition of strong alkali and weak alkali, resulting in an increase in viscosity at the beginning of the process. Eventually, the viscosity retention rate of the three discussed flooding systems all exceeds 80%,. In addition to that, the ASP and SP combination flooding systems all show excellent adsorption resistance as the interfacial tension remains at ultra-low level even after 4–5 repetitive adsorption test.
4. Overall, the discussed combination flooding systems have all achieved positive oil displacement effect. The weak alkali ASP system has the best average chemical flooding recovery rate with 33.83%, a three percentage points increase on the 31.34% recovery rate for the SP system and over seven percentage points more than the 26.71% recovery rate of the strong alkali ASP system. In terms of oil recovery, the weak alkali ASP flooding is the highest, the SP flooding ranks the second, and the strong alkali ASP flooding is the worst. During subsequent water flooding of the strong alkali ASP system, the pressure decreases significantly due to its weak emulsification.

## Supporting information

S1 File Data. (DOC)
