# Supplementary material for: Flooding performance evaluation of alkyl aryl sulfonate in various alkaline environments
Source: PLoS One. 2019 Sep 12;14(9):e0219627. doi: 10.1371/journal.pone.0219627 (PMC6742392; doi:10.1371/journal.pone.0219627)
Supplement: S1 File — (DOC) [file pone.0219627.s001.doc]

Dear editor:

All data underlying the findings described in my manuscript fully available without restriction at the time of publication. I guarantee that all the data in the manuscript are obtained from authentic experiments. The experiment has considerable accuracy and repeatability.

Experimental schemes for oil displacement were developed by flooding schemes (table 1) as following table 1-1 to table 1-6.

Table 1-1 Displacement process data of the No. 11 flooding scheme

| Core  No. | Length | Cross  -Sectional area | | Pore Volume | | Porosity | | Satura-tion of Oil | | Satura-tion | Oil by Water Flooding | | Recovery of Water Flooding | | Gas Permea-bility | | Water Measures Permea-  bility | |
| --- | --- | --- | --- | --- | --- | --- | --- | --- | --- | --- | --- | --- | --- | --- | --- | --- | --- | --- |
|  | (cm) | (cm^2^) | | (mL) | | (%) | | (mL) | | (%) | (mL) | | (%) | | (mD) | | (mD) | |
| 1811-1 | 30.5 | 20.43 | | 128 | | 20.54 | | 81.4 | | 63.59 | 30.20 | | 37.10 | | 500 | | 204 | |
| Scheme | Water Flooding + 0.3PV SP Flooding + 0.2PV Polymer Flooding + Subsequent Water Flooding | | | | | | | | | | | | | | | | | |
| No. | Differential Pressure | | Volume | Water | Oil | | Cumulative Oil | | Recovery | | | Moisture Content | | Cumulative Volume | | PV | | Note |
|  | /MPa | | /mL | /mL | /mL | | /mL | | /% | | | /% | | /mL | |  |  |  |
| SP Flooding | 0.068 | | 10 | 10 | 0 | | 0.000 | | 0.00 | | | 100.00 | | 10.00 | | 0.078 | |  |
|  | 0.124 | | 10 | 10 | 0 | | 0.000 | | 0.00 | | | 100.00 | | 20.00 | | 0.156 | |  |
|  | 0.157 | | 10 | 7.6 | 2.4 | | 2.400 | | 2.95 | | | 76.00 | | 30.00 | | 0.234 | |  |
|  | 0.173 | | 8.4 | 3.4 | 5.0 | | 7.400 | | 9.09 | | | 40.48 | | 38.40 | | 0.300 | |  |
| Polymer Flooding | 0.200 | | 9.3 | 5.6 | 3.7 | | 11.100 | | 13.64 | | | 60.22 | | 47.70 | | 0.373 | |  |
|  | 0.228 | | 10 | 6.5 | 3.5 | | 14.600 | | 17.94 | | | 65.00 | | 57.70 | | 0.451 | |  |
|  | 0.247 | | 6.6 | 4.5 | 2.1 | | 16.700 | | 20.52 | | | 68.18 | | 64.30 | | 0.502 | |  |
| Sub-  sequent Water Flooding | 0.279 | | 10 | 7.2 | 2.8 | | 19.500 | | 23.96 | | | 72.00 | | 74.30 | | 0.580 | |  |
|  | 0.260 | | 10 | 7.4 | 2.6 | | 22.100 | | 27.15 | | | 74.00 | | 84.30 | | 0.659 | |  |
|  | 0.191 | | 10 | 8.4 | 1.6 | | 23.700 | | 29.12 | | | 84.00 | | 94.30 | | 0.737 | | Emulsion |
|  | 0.128 | | 10 | 8.8 | 1.2 | | 24.900 | | 30.59 | | | 88.00 | | 104.30 | | 0.815 | | Emulsion |
|  | 0.089 | | 10 | 9.8 | 0.2 | | 25.100 | | 30.84 | | | 98.00 | | 114.30 | | 0.893 | | Emulsion |
|  | 0.074 | | 10 | 10 | 0 | | 25.100 | | 30.84 | | | 100.00 | | 124.30 | | 0.971 | |  |

(a) Phase of chemical injection	(b) Phase of water flooding

Fig. 1-1 Macrophotograph of chemical flooding in the No. 11 flooding scheme

Table 1-2 Displacement process data of the No. 21 flooding scheme

| Core  No. | Length | Cross  -sectional Area | | Pore Volume | | Porosity | | Satura-tion of Oil | | Satura-tion | Oil by Water Flooding | | Recovery of Water Flooding | | Gas Permea-bility | | Water Measures Permea-  bility | |
| --- | --- | --- | --- | --- | --- | --- | --- | --- | --- | --- | --- | --- | --- | --- | --- | --- | --- | --- |
|  | (cm) | (cm^2^) | | (mL) | | (%) | | (mL) | | (%) | (mL) | | (%) | | (mD) | | (mD) | |
| 1811-2 | 30.5 | 20.48 | | 127.8 | | 20.46 | | 83.2 | | 65.10 | 31.70 | | 38.10 | | 500 | | 204 | |
| Scheme | Water Flooding + 0.3 PV Weak Alkali ASP Flooding + 0.2 PV Polymer Flooding + Subsequent Water Flooding | | | | | | | | | | | | | | | | | |
| No. | Differential Pressure | | Volume | Water | Oil | | Cumulative Oil | | Recovery | | | Moisture Content | | Cumulative Volume | | PV | | Note |
|  | /MPa | | /mL | /mL | /mL | | /mL | | /% | | | /% | | /mL | |  |  |  |
| ASP Flooding | 0.207 | | 10 | 10 | 0 | | 0.000 | | 0.00 | | | 100.00 | | 10.00 | | 0.078 | |  |
|  | 0.235 | | 10 | 10 | 0 | | 0.000 | | 0.00 | | | 100.00 | | 20.00 | | 0.156 | |  |
|  | 0.250 | | 10 | 8.1 | 1.9 | | 1.900 | | 2.28 | | | 81.00 | | 30.00 | | 0.235 | |  |
|  | 0.245 | | 8.4 | 2.8 | 5.6 | | 7.500 | | 9.01 | | | 33.33 | | 38.40 | | 0.300 | |  |
| Polymer Flooding | 0.256 | | 10.4 | 5.6 | 4.8 | | 12.300 | | 14.78 | | | 53.85 | | 48.80 | | 0.382 | |  |
|  | 0.282 | | 10 | 6.0 | 4.0 | | 16.300 | | 19.59 | | | 60.00 | | 58.80 | | 0.460 | |  |
|  | 0.292 | | 5.2 | 3.2 | 2.0 | | 18.300 | | 22.00 | | | 61.54 | | 64.00 | | 0.501 | |  |
| Sub-  sequent Water Flooding | 0.288 | | 10 | 6.9 | 3.1 | | 21.400 | | 25.72 | | | 69.00 | | 74.00 | | 0.579 | |  |
|  | 0.299 | | 10.1 | 7.3 | 2.8 | | 24.200 | | 29.09 | | | 72.28 | | 84.10 | | 0.658 | |  |
|  | 0.281 | | 10 | 8.6 | 1.4 | | 25.600 | | 30.77 | | | 86.00 | | 94.10 | | 0.736 | | Emulsion |
|  | 0.242 | | 10 | 8.9 | 1.1 | | 26.700 | | 32.09 | | | 89.00 | | 104.10 | | 0.815 | | Emulsion |
|  | 0.239 | | 10 | 9.5 | 0.5 | | 27.200 | | 32.69 | | | 95.00 | | 114.10 | | 0.893 | | Emulsion |
|  | 0.236 | | 10 | 9.8 | 0.2 | | 27.400 | | 32.93 | | | 98.00 | | 124.10 | | 0.971 | |  |

(a) Phase of chemical injection	(b) Phase of water flooding

Fig. 1-2 Macrophotograph of chemical flooding in the No. 21 flooding scheme

Table 1-3 Displacement process data of the No. 31 flooding scheme

| Core  No. | Length | Cross  -sectional Area | | Pore Volume | | Porosity | | Satura-tion of Oil | | Satura-tion | Oil by Water Flooding | | Recovery of Water Flooding | | Gas Permea-bility | | Water Measures Permea-  bility | |
| --- | --- | --- | --- | --- | --- | --- | --- | --- | --- | --- | --- | --- | --- | --- | --- | --- | --- | --- |
|  | (cm) | (cm^2^) | | (mL) | | (%) | | (mL) | | (%) | (mL) | | (%) | | (mD) | | (mD) | |
| 1811-3 | 30.5 | 20.48 | | 128.6 | | 20.59 | | 83.4 | | 64.85 | 31.20 | | 37.41 | | 500 | | 215 | |
| Scheme | Water Flooding +0.3 PV Strong Alkali ASP Flooding + 0.2 PV Polymer Flooding + Subsequent Water Flooding | | | | | | | | | | | | | | | | | |
| No. | Differential Pressure | | Volume | Water | Oil | | Cumulative Oil | | Recovery | | | Moisture Content | | Cumulative Volume | | PV | | Note |
|  | /MPa | | /mL | /mL | /mL | | /mL | | /% | | | /% | | /mL | |  |  |  |
| ASP Flooding | 0.138 | | 10 | 10 | 0 | | 0.000 | | 0.00 | | | 100.00 | | 10.00 | | 0.078 | |  |
|  | 0.167 | | 10 | 10 | 0 | | 0.000 | | 0.00 | | | 100.00 | | 20.00 | | 0.156 | |  |
|  | 0.237 | | 10 | 8.2 | 1.8 | | 1.800 | | 2.16 | | | 82.00 | | 30.00 | | 0.233 | |  |
|  | 0.257 | | 8.6 | 3.0 | 5.6 | | 7.400 | | 8.87 | | | 34.88 | | 38.60 | | 0.300 | |  |
| Polymer Flooding | 0.288 | | 10 | 6.1 | 3.9 | | 11.300 | | 13.55 | | | 61.00 | | 48.60 | | 0.378 | |  |
|  | 0.279 | | 10.2 | 6.6 | 3.6 | | 14.900 | | 17.87 | | | 64.71 | | 58.80 | | 0.457 | |  |
|  | 0.299 | | 5.7 | 3.9 | 1.8 | | 16.700 | | 20.02 | | | 68.42 | | 64.50 | | 0.502 | |  |
| Sub-  sequent Water Flooding | 0.321 | | 10 | 7.5 | 2.5 | | 19.200 | | 23.02 | | | 75.00 | | 74.50 | | 0.579 | |  |
|  | 0.315 | | 10 | 8.1 | 1.9 | | 21.100 | | 25.30 | | | 81.00 | | 84.50 | | 0.657 | | Emulsion |
|  | 0.205 | | 10.1 | 9.2 | 0.9 | | 22.000 | | 26.38 | | | 91.09 | | 94.60 | | 0.736 | | Emulsion |
|  | 0.165 | | 10 | 9.6 | 0.4 | | 22.400 | | 26.86 | | | 96.00 | | 104.60 | | 0.813 | | Emulsion |
|  | 0.155 | | 10 | 9.8 | 0.2 | | 22.600 | | 27.10 | | | 98.00 | | 114.60 | | 0.891 | |  |

(a) Phase of chemical injection	(b) Phase of water flooding

Fig. 1-3 Macrophotograph of chemical flooding in the No. 31 flooding scheme

Table 1-4 Displacement process data of the No. 12 flooding scheme

| Core  No. | Length | Cross  -Sectional area | | Pore Volume | | Porosity | | Satura-tion of Oil | | Satura-tion | Oil by Water Flooding | | Recovery of Water Flooding | | Gas Permea-bility | | Water Measures Permea-  bility | |
| --- | --- | --- | --- | --- | --- | --- | --- | --- | --- | --- | --- | --- | --- | --- | --- | --- | --- | --- |
|  | (cm) | (cm^2^) | | (mL) | | (%) | | (mL) | | (%) | (mL) | | (%) | | (mD) | | (mD) | |
| 1811-4 | 30.5 | 20.7 | | 124 | | 19.64 | | 84.2 | | 67.90 | 31.20 | | 37.05 | | 500 | | 201 | |
| Scheme | Water Flooding + 0.3PV SP Flooding + 0.2PV Polymer Flooding + Subsequent Water Flooding | | | | | | | | | | | | | | | | | |
| No. | Differential Pressure | | Volume | Water | Oil | | Cumulative Oil | | Recovery | | | Moisture Content | | Cumulative Volume | | PV | | Note |
|  | /MPa | | /mL | /mL | /mL | | /mL | | /% | | | /% | | /mL | |  |  |  |
| SP Flooding | 0.113 | | 10 | 10 | 0 | | 0.000 | | 0.00 | | | 100.00 | | 10.00 | | 0.081 | |  |
|  | 0.182 | | 10 | 10 | 0 | | 0.000 | | 0.00 | | | 100.00 | | 20.00 | | 0.161 | |  |
|  | 0.232 | | 10 | 7.5 | 2.5 | | 2.500 | | 2.97 | | | 75.00 | | 30.00 | | 0.242 | |  |
|  | 0.247 | | 7.4 | 3.0 | 4.4 | | 6.900 | | 8.19 | | | 40.54 | | 37.40 | | 0.302 | |  |
| Polymer Flooding | 0.260 | | 10 | 6.3 | 3.7 | | 10.600 | | 12.59 | | | 63.00 | | 47.40 | | 0.382 | |  |
|  | 0.277 | | 10 | 6.4 | 3.6 | | 14.200 | | 16.86 | | | 64.00 | | 57.40 | | 0.463 | |  |
|  | 0.284 | | 5.2 | 2.8 | 2.4 | | 16.600 | | 19.71 | | | 53.85 | | 62.60 | | 0.505 | |  |
| Sub-  sequent Water Flooding | 0.257 | | 10.1 | 7.0 | 3.1 | | 19.700 | | 23.40 | | | 69.31 | | 72.70 | | 0.586 | |  |
|  | 0.249 | | 10 | 7.0 | 3.0 | | 22.700 | | 26.96 | | | 70.00 | | 82.70 | | 0.667 | |  |
|  | 0.193 | | 10 | 8.0 | 2.0 | | 24.700 | | 29.33 | | | 80.00 | | 92.70 | | 0.748 | | Emulsion |
|  | 0.145 | | 10 | 8.8 | 1.2 | | 25.900 | | 30.76 | | | 88.00 | | 102.70 | | 0.828 | | Emulsion |
|  | 0.108 | | 10 | 9.3 | 0.7 | | 26.600 | | 31.59 | | | 93.00 | | 112.70 | | 0.909 | | Emulsion |
|  | 0.089 | | 10 | 9.8 | 0.2 | | 26.800 | | 31.83 | | | 98.00 | | 122.70 | | 0.990 | |  |

(a) Phase of chemical injection	(b) Phase of water flooding

Fig. 1-4 Macrophotograph of chemical flooding in the No. 12 flooding scheme

Table 1-5 Displacement process data of the No. 22 flooding scheme

| Core  No. | Length | Cross  -sectional Area | | Pore Volume | | Porosity | | Satura-tion of Oil | | Satura-tion | Oil by Water Flooding | | Recovery of Water Flooding | | Gas Permea-bility | | Water Measures Permea-  bility | |
| --- | --- | --- | --- | --- | --- | --- | --- | --- | --- | --- | --- | --- | --- | --- | --- | --- | --- | --- |
|  | (cm) | (cm^2^) | | (mL) | | (%) | | (mL) | | (%) | (mL) | | (%) | | (mD) | | (mD) | |
| 1811-5 | 30.5 | 20.43 | | 125.8 | | 20.19 | | 81.2 | | 64.55 | 30.70 | | 37.81 | | 500 | | 150 | |
| Scheme | Water Flooding + 0.3 PV Weak Alkali ASP Flooding + 0.2 PV Polymer Flooding + Subsequent Water Flooding | | | | | | | | | | | | | | | | | |
| No. | Differential Pressure | | Volume | Water | Oil | | Cumulative Oil | | Recovery | | | Moisture Content | | Cumulative Volume | | PV | | Note |
|  | /MPa | | /mL | /mL | /mL | | /mL | | /% | | | /% | | /mL | |  |  |  |
| ASP Flooding | 0.162 | | 10 | 10 | 0 | | 0.000 | | 0.00 | | | 100.00 | | 10.00 | | 0.079 | |  |
|  | 0.205 | | 10 | 10 | 0 | | 0.000 | | 0.00 | | | 100.00 | | 20.00 | | 0.159 | |  |
|  | 0.218 | | 10 | 7.9 | 2.1 | | 2.100 | | 2.59 | | | 79.00 | | 30.00 | | 0.238 | |  |
|  | 0.221 | | 8.0 | 2.6 | 5.4 | | 7.500 | | 9.24 | | | 32.50 | | 38.00 | | 0.302 | |  |
| Polymer Flooding | 0.244 | | 10 | 5.7 | 4.3 | | 11.800 | | 14.53 | | | 57.00 | | 48.00 | | 0.382 | |  |
|  | 0.275 | | 10.2 | 6.2 | 4.0 | | 15.800 | | 19.46 | | | 60.78 | | 58.20 | | 0.463 | |  |
|  | 0.288 | | 5.0 | 3.2 | 1.8 | | 17.600 | | 21.67 | | | 64.00 | | 63.20 | | 0.502 | |  |
| Sub-  sequent Water Flooding | 0.291 | | 10 | 6.9 | 3.1 | | 20.700 | | 25.49 | | | 69.00 | | 73.20 | | 0.582 | |  |
|  | 0.307 | | 10 | 6.9 | 3.1 | | 23.800 | | 29.31 | | | 69.00 | | 83.20 | | 0.661 | |  |
|  | 0.256 | | 10 | 7.4 | 2.6 | | 26.400 | | 32.51 | | | 74.00 | | 93.20 | | 0.741 | | Emulsion |
|  | 0.202 | | 10 | 8.8 | 1.2 | | 27.600 | | 33.99 | | | 88.00 | | 103.20 | | 0.820 | | Emulsion |
|  | 0.196 | | 10.1 | 9.7 | 0.4 | | 28.000 | | 34.48 | | | 96.04 | | 113.30 | | 0.901 | | Emulsion |
|  | 0.204 | | 10 | 9.8 | 0.2 | | 28.200 | | 34.73 | | | 98.00 | | 123.30 | | 0.980 | |  |

(a) Phase of chemical injection	(b) Phase of water flooding

Fig. 1-5 Macrophotograph of chemical flooding in the No. 22 flooding scheme

Table 1-6 Displacement process data of the No. 32 flooding scheme

| Core  No. | Length | Cross  -sectional Area | | Pore Volume | | Porosity | | Satura-tion of Oil | | Satura-tion | Oil by Water Flooding | | Recovery of Water Flooding | | Gas Permea-bility | | Water Measures Permea-  bility | |
| --- | --- | --- | --- | --- | --- | --- | --- | --- | --- | --- | --- | --- | --- | --- | --- | --- | --- | --- |
|  | (cm) | (cm^2^) | | (mL) | | (%) | | (mL) | | (%) | (mL) | | (%) | | (mD) | | (mD) | |
| 1811-6 | 30.5 | 20.79 | | 127.2 | | 20.06 | | 80.2 | | 63.05 | 30.70 | | 38.28 | | 500 | | 139 | |
| Scheme | Water Flooding +0.3 PV Strong Alkali ASP Flooding + 0.2 PV Polymer Flooding + Subsequent Water Flooding | | | | | | | | | | | | | | | | | |
| No. | Differential Pressure | | Volume | Water | Oil | | Cumulative Oil | | Recovery | | | Moisture Content | | Cumulative Volume | | PV | | Note |
|  | /MPa | | /mL | /mL | /mL | | /mL | | /% | | | /% | | /mL | |  |  |  |
| ASP Flooding | 0.168 | | 10 | 10 | 0 | | 0.000 | | 0.00 | | | 100.00 | | 10.00 | | 0.079 | |  |
|  | 0.206 | | 10 | 10 | 0 | | 0.000 | | 0.00 | | | 100.00 | | 20.00 | | 0.157 | |  |
|  | 0.265 | | 10 | 9.1 | 0.9 | | 0.900 | | 1.12 | | | 91.00 | | 30.00 | | 0.236 | |  |
|  | 0.316 | | 8.2 | 3.0 | 5.2 | | 6.100 | | 7.61 | | | 36.59 | | 38.20 | | 0.300 | |  |
| Polymer Flooding | 0.329 | | 10.2 | 6.3 | 3.9 | | 10.000 | | 12.47 | | | 61.76 | | 48.40 | | 0.381 | |  |
|  | 0.315 | | 10 | 6.5 | 3.5 | | 13.500 | | 16.83 | | | 65.00 | | 58.40 | | 0.459 | |  |
|  | 0.336 | | 5.5 | 3.7 | 1.8 | | 15.300 | | 19.08 | | | 67.27 | | 63.90 | | 0.502 | |  |
| Sub-  sequent Water Flooding | 0.370 | | 10 | 7.5 | 2.5 | | 17.800 | | 22.19 | | | 75.00 | | 73.90 | | 0.581 | |  |
|  | 0.357 | | 10 | 8.2 | 1.8 | | 19.600 | | 24.44 | | | 82.00 | | 83.90 | | 0.660 | | Emulsion |
|  | 0.290 | | 10 | 9.2 | 0.8 | | 20.400 | | 25.44 | | | 92.00 | | 93.90 | | 0.738 | | Emulsion |
|  | 0.211 | | 10 | 9.5 | 0.5 | | 20.900 | | 26.06 | | | 95.00 | | 103.90 | | 0.817 | | Emulsion |
|  | 0.171 | | 10 | 9.8 | 0.2 | | 21.100 | | 26.31 | | | 98.00 | | 113.90 | | 0.895 | |  |

(a) Phase of chemical injection	(b) Phase of water flooding

Fig. 1-6 Macrophotograph of chemical flooding in the No. 32 flooding scheme
